# Hypercapnia increases ACE2 expression and pseudo-SARS-CoV-2 entry in bronchial epithelial cells by augmenting cellular cholesterol

**DOI:** 10.3389/fimmu.2023.1251120

**Published:** 2023-10-12

**Authors:** Fei Chen, Aiko Matsuda, G. R. Scott Budinger, Peter H. S. Sporn, S. Marina Casalino-Matsuda

**Affiliations:** ^1^ Division of Pulmonary and Critical Care Medicine, Feinberg School of Medicine, Northwestern University, Chicago, IL, United States; ^2^ Research Service, Jesse Brown Veterans Affairs Medical Center, Chicago, IL, United States

**Keywords:** hypercapnia, COVID-19, ACE2, SARS-CoV-2, cholesterol, statins

## Abstract

Patients with chronic lung disease, obesity, and other co-morbid conditions are at increased risk of severe illness and death when infected with severe acute respiratory syndrome coronavirus 2 (SARS-CoV-2). Hypercapnia, the elevation of CO_2_ in blood and tissue, commonly occurs in patients with severe acute and chronic lung disease, including those with pulmonary infections, and is also associated with high mortality risk. We previously reported that hypercapnia increases viral replication and mortality of influenza A virus infection in mice. We have also shown that culture in elevated CO_2_ upregulates expression of cholesterol synthesis genes in primary human bronchial epithelial cells. Interestingly, factors that increase the cholesterol content of lipid rafts and lipid droplets, platforms for viral entry and assembly, enhance SARS-CoV-2 infection. In the current study, we investigated the effects of hypercapnia on ACE2 expression and entry of SARS-CoV-2 pseudovirus (p-SARS-CoV-2) into airway epithelial cells. We found that hypercapnia increased ACE2 expression and p-SARS-CoV-2 uptake by airway epithelium in mice, and in cultured VERO and human bronchial epithelial cells. Hypercapnia also increased total cellular and lipid raft-associated cholesterol in epithelial cells. Moreover, reducing cholesterol synthesis with inhibitors of sterol regulatory element binding protein 2 (SREBP2) or statins, and depletion of cellular cholesterol, each blocked the hypercapnia-induced increases in ACE2 expression and p-SARS-CoV-2 entry into epithelial cells. Cigarette smoke extract (CSE) also increased ACE2 expression, p-SARS-CoV-2 entry and cholesterol accumulation in epithelial cells, an effect not additive to that of hypercapnia, but also inhibited by statins. These findings reveal a mechanism that may account, in part, for poor clinical outcomes of SARS-CoV-2 infection in patients with advanced lung disease and hypercapnia, and in those who smoke cigarettes. Further, our results suggest the possibility that cholesterol-lowering therapies may be of particular benefit in patients with hypercapnia when exposed to or infected with SARS-CoV-2.

## Graphical Abstract

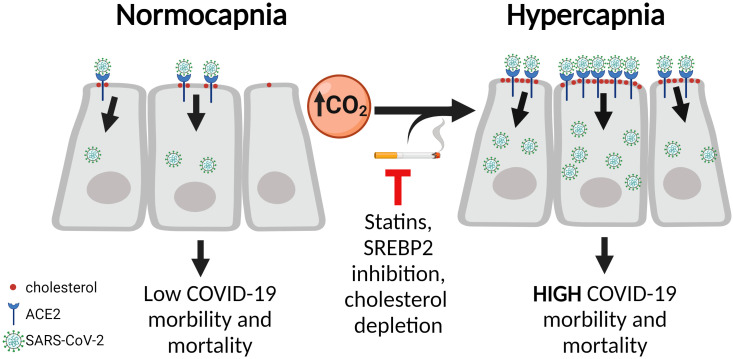

## 1 Introduction

Severe acute respiratory syndrome coronavirus 2 (SARS-CoV-2), the betacoronavirus that causes coronavirus disease 19 (COVID-19), has been responsible for approximately 770 million reported cases and nearly 7 million reported deaths worldwide, as of August 2023 (1). Patients with pre-existing conditions including chronic obstructive pulmonary diseases (COPD) and cigarette smokers, are at higher risk of severe illness and death from COVID-19 (2–4). Infection with SARS-CoV-2 depends on expression of angiotensin-converting enzyme 2 (ACE2), which binds the viral spike protein receptor-binding domain (RBD) and mediates entry of the virus into host cells (5). Several studies have shown that ACE2 expression is increased in bronchial epithelium of patients with comorbidities associated with severe COVID-19, a mechanism that would be expected to enhance SARS-CoV-2 infection in the airways (6–9). Hypercapnia, the elevation of the partial pressure of carbon dioxide (CO_2_) in blood and tissue, commonly develops in severe acute and chronic lung diseases, including advanced COPD, and is associated with frequent pulmonary infections, which can be fatal (10–14). In a multicenter study of patients hospitalized in the ICU for severe COVID-19, those with hypercapnia had more severe lung injury, spent more time on mechanical ventilation, and stayed longer in the ICU than those who were normocapnic (15). Furthermore, in a mouse model of influenza infection, we previously reported that hypercapnia increased expression of viral proteins, viral replication, lung injury, and mortality following inoculation with influenza A virus (16, 17).

In a transcriptomic profiling study of cultured primary human bronchial epithelial (HBE) cells, we found that hypercapnia upregulated expression of cholesterol biosynthesis genes including 3-hydroxy-3-methylglutaryl-CoA synthase 1 (HMGCS1) and downregulated ATP-binding cassette (ABC) transporters, which promote cholesterol efflux (18). These genes are regulated by the transcription factor sterol-regulatory element binding protein 2 (SREBP2), master regulator of cholesterol synthesis and transport genes (19). Interestingly, factors that increase the cholesterol content of lipid rafts and lipid droplets, which serve as platforms for viral entry and assembly (20–22), increase SARS-CoV-2 infection in cultured cells. Moreover, retrospective and cohort studies suggest that chronic therapy with statins, which inhibit cholesterol synthesis, protects against adverse outcomes of COVID-19 (23–26).

In the current study, we investigated the effects of hypercapnia on ACE2 expression and entry of SARS-CoV-2 pseudovirus (p-SARS-CoV-2) in airway epithelial cells. We observed that hypercapnia increased ACE2 expression and p-SARS-CoV-2 uptake by airway epithelium in mice, and in cultured VERO and HBE cells. Elevated CO_2_ also increased total cellular and lipid raft cholesterol content. Moreover, inhibition of cholesterol synthesis with SREBP2 inhibitors or statins, and pharmacological depletion of cellular cholesterol, both blocked the hypercapnia-induced increases in ACE2 expression and p-SARS-CoV-2 entry into epithelial cells. Cigarette smoke extract (CSE) also increased ACE2 expression, p-SARS-CoV-2 entry and cholesterol accumulation in epithelial cells, an effect not additive to that of hypercapnia, but also inhibited by statins.

## 2 Materials and methods

### 2.1 Materials

All materials were purchased from Sigma-Aldrich, unless otherwise specified.

### 2.2 Mice

Six- to ten-week-old C57BL/6 mice from The Jackson Laboratory were used. Experiments were performed according to a protocol approved by the Institutional Animal Care and Use Committee of Northwestern University and according to National Institutes of Health guidelines for the use of rodents.

### 2.3 Murine hypercapnia exposure

As previously described (27), mice were housed in a BioSpherix A environmental chamber (BioSpherix) with ProOx C21 O_2_ and CO_2_ controllers (BioSpherix), that maintain an environment of 10% CO_2_/21% O_2_/69% N_2_ (normoxic hypercapnia). Simultaneously, age-matched mice were maintained in ambient air and used as controls. Betulin (5 mg/kg body weight) was administrated 3 days before and on the day that hypercapnia exposure began.

### 2.4 Cells

Primary human bronchial epithelial (HBE) cells (LONZA) were seeded as passage 1 (P1) into 100 mm dishes on PneumaCult-Ex Plus media (StemCell) and incubated at 37°C, 5% CO_2_. Once cells reached 70–80% confluency, they were dissociated using Animal Component-Free Cell Dissociation Kit (StemCell) and seeded on 12 mm Transwells (Corning) coated with 0.3 mg/mL Collagen type IV from human placenta (Sigma-Aldrich). Upon reaching confluency, the apical medium was removed to obtain an air liquid interface (ALI), and the basal medium replaced with PneumaCult-ALI medium (StemCell). Medium was changed every second day and apical surfaces washed with PBS (Ca^2+^, Mg^2+^) twice per week. Cultures were used for experiments after reaching full differentiation (∼ 3-4 wk on air) as assessed by visual confirmation of beating cilia and mucus (28). BEAS-2B cells, a SV-40-transformed human bronchial epithelial cell line (ATCC CRL-9609), were maintained in DMEM:F12, 5% FBS medium while VERO (ATCC, CRL-1586) were grown on EMEM, 10% FBS. All media contained penicillin (100 U/ml) and streptomycin (100 μg/ml).

### 2.5 Exposure of cells to normocapnia, hypercapnia and cigarette smoke extract

Cells were exposed to normocapnia or normoxic hypercapnia. Normocapnia is defined as humidified 5% CO_2_ (PCO_2_ 36 mmHg)/95% air, at 37°C (standard incubator atmosphere). Normoxic hypercapnia entailed 15% CO_2_ (PCO_2_ 108 mmHg)/21% O_2_/64% N_2_ in an environmental chamber (C-174, Biospherix) contained within the same incubator where control cultures were simultaneously exposed to normocapnia. In selected experiments, pH at 7.4 in 15% CO_2_ or 7.2 in 5% CO_2_ was maintained adding Tris-HCl and Tris base to the media and measured with a pHOx Plus blood gas analyzer (Nova Biomedical). Before addition to cultures, media were pre-saturated with 5% or 15% CO_2_. Cells were also exposed to cigarette smoke extract (CSE, 1 μg/ml, Murty Pharmaceuticals) during culture in normocapnia or hypercapnia.

### 2.6 Infection with pseudo-SARS-CoV-2

Cells were pre-exposed to normocapnia or hypercapnia in the presence or the absence of CSE for 2 days, pseudo-SARS-CoV-2 virus (p-SARS-CoV-2, Montana Molecular, 1 x 10^8^ PV/ml) was added, and cells were cultured for an additional day in normocapnia or hypercapnia, respectively. The p-SARS-CoV-2 virus is a baculovirus pseudo-typed with SARS-CoV-2 spike protein that does not replicate but delivers a genetically encoded green-fluorescent reporter to infected host cells and is safe to use with no risk of infection to laboratory personnel. Nuclei were labeled with DAPI. Cells were imaged using fluorescence microscopy (3 fields/condition from at least 4 independent experiments) and the percentage of pseudo-SARS-CoV-2 positive cells was assessed using CellProfiler™ software (29).

### 2.7 Immunoblotting

The presence of indicated proteins in cell homogenates were assessed by immunoblotting, as before (16), using the following antibodies: anti-ACE2 (GeneTex, SN0754) and anti-βactin (Abcam, ab8226) followed by HRP-conjugated (1:5000, CellSignal) or IRDye (1:10,000, LI-COR) secondary antibodies, respectively. Signals were captured with a LI-COR Odyssey Fc Imager and analyzed by densitometry using ImageStudio™ software (LI-COR). Images are representative of 3 or more independent experiments.

### 2.8 Immunofluorescence microscopy in tissue sections and cell cultures

After euthanasia, mouse lungs were perfused with HBSS via the right ventricle and a 20-gauge angiocath was sutured into the trachea. Lungs and heart were removed *en bloc*, and lungs were inflated at a pressure of 16 cm H_2_O with 0.8 ml of formalin. Tissue was embedded in paraffin and 5-μm sections were then deparaffinized with xylene, rehydrated by using graded ethanol, and subjected to antigen retrieval using sodium citrate buffer (10 mM, pH 6.0), as before (16). Tissues were labeled with anti-ACE2 (GeneTex, SN0754), anti ABCA1 (Thermo, MA5-16026) or anti-SREBP2 (Novus, NBP1-54446SS) antibodies followed by Alexa-conjugated secondary antibodies (1 μg/ml). Cells were fixed with 4% PFA for 15 min or methanol (-20°C for 5 min), permeabilized with 0.1% Triton X100 for 5 min, and labeled with anti-ACE2 (Abcam, ab15348 or GeneTex, SN0754) or anti-SREBP2 (Novus, NBP1-54446SS) antibodies followed by Alexa-conjugated secondary antibodies. Cells were co-labeled with acetylated tubulin (cilliated cell marker) using anti-acetylated-tubulin (T7451) antibody, followed by Alexa-conjugated antibody (1 μg/ml). In all cases, DAPI was used to visualize nuclei, and Gel/Mount (Biomeda) was used to mount the slides. Of note, mouse or rabbit IgGs were used as a nonimmune staining control that was negative for all protocols. Images were obtained using the same exposure time for all samples from a given experimental set using Axiovert 200M Fluorescence Microscope (Zeiss). Exposure time was selected based on the brightest stained sample to avoid saturation, and used for all other samples in the set, resulting in equal subtraction of background autofluorescence from all sets. Tissue images are representative of at least 3 independent experiments. Cells were also imaged using fluorescence microscopy (3 fields/condition from at least 4 independent experiments), and fluorescence intensity was quantified using NIH ImageJ software. Nuclei/field were assessed with CellProfiler cell image analysis software. ACE2 fluorescence intensity was expressed in arbitrary units (AU)/cell.

### 2.9 Analysis of transcriptomic dataset

We included genes obtained by Gene Ontology (GO) analysis of biological processes for cholesterol efflux and biosynthesis from our previous published study (18) investigating the transcriptomic response to hypercapnia in human bronchial epithelial cells (GSE110362). We determined transcriptional changes induced by elevated CO_2_ in HBE cells differentiated at ALI were exposed to hypercapnia for 24 h or maintained in normocapnia as a control using Affymetrix GeneChip Hybridization. Relative gene expression was represented as a heat map.

### 2.10 Cell cholesterol and lipid rafts

Cells were lysed using RIPA buffer and cholesterol was measured using Amplex red assay following manufacturer’s protocol. Cholesterol concentration was expressed as µg/mg protein. In addition, cells were incubated with filipin III (1 µg/ml), cholera toxin B, or LipidSpot 488 (Biotium) at 37°C for 30 min, to stain total cholesterol, ganglioside GM1 in lipid rafts, or lipid droplets (LD), respectively. Nuclei were stained with DAPI, then imaged by fluorescence microscopy.

### 2.11 Quantitative real-time PCR

RNeasy Mini Kit (Qiagen) was used to extract RNA that was reverse transcribed with an iScript cDNA Synthesis Kit (Bio-Rad), as before (16). PrimeTime™ Predesigned qPCR Assays FAM-labeled primer/probes sets Hs.PT.58.27645939 for ACE2 and Hs.PT.58v.18759587 for the housekeeping gene B2M were used for amplification using the CFX Connect Real-Time System (Bio-Rad). ACE2 gene expression was normalized to B2M. Comparative CT method (ΔΔCT) was used to assess mRNA relative expression, as before (16).

### 2.12 Statistical analysis

Data were analyzed using Prism 9.0 (GraphPad). Student’s t test and Levene’s test were used to determine differences between two groups and homogeneity of variances, respectively. ANOVA followed by Sidak’s multiple comparisons test was used to analyze differences between multiple groups. In all analyses, p < 0.05 was considered significant.

## 3 Results

### 3.1 Hypercapnia increases ACE2 protein expression in murine airway epithelium, in human bronchial epithelial cells, and in VERO cells

**Figure 1:**
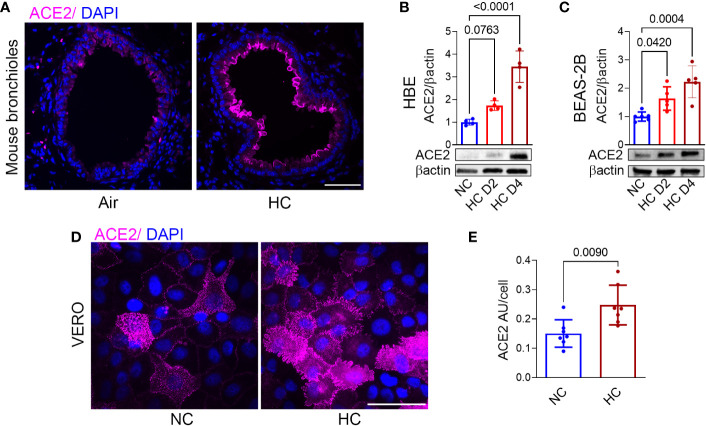
Hypercapnia increases ACE2 protein expression in murine bronchial epithelium, human bronchial epithelial cells, and VERO cells. ACE2 protein expression was assessed by immunofluorescence microscopy (IF) in lungs of mice breathing ambient air or normoxic hypercapnia (HC) for 7 days **(A)**, in differentiated human bronchial epithelial (HBE) cells **(B)** and BEAS-2B cells **(C)** cultured in normocapnia (NC) or HC for 2 (D2) or 4 (D4) days, and by IF in VERO cells cultured in NC or HC for 3 days **(D)**. ACE2 expression in VERO cells was quantified as relative fluorescence intensity per cell and expressed as arbitrary units (AU)/cell **(E)**. Nuclei were stained with DAPI. In **(A, D)**, scale bars = 50 µM. For immunoblots, β-actin was used as loading control. All data are means ± SD. P values for comparisons between groups are shown **(B, C, E)**.

To investigate the effects of hypercapnia on ACE2 protein expression *in vivo*, mice were exposed to 10% CO_2_/21% O_2_/69% N_2_ (normoxic hypercapnia) for 7, 14 and 21 days, or ambient air as control. We and our colleagues have previously shown that exposure of mice 10% CO_2_ for up to 21 days causes no obvious signs of distress or overt changes in the animals’ appearance, and that they gain weight normally in comparison with air-exposed control mice (16, 27, 30, 31). In addition, we showed that exposure to normoxic hypercapnia for up to 7 days caused no histopathologic lung injury (16). In the current study, hypercapnia increased ACE2 expression in the bronchi of mice exposed to 10% CO_2_ for 7, 14 and 21 days (**Figure 1A** and **Supplementary Figure 1**). SARS-CoV-2 does not replicate in wild-type mice, due to poor binding to mouse ACE2 (32). Therefore, to study the impact of hypercapnia on SARS-CoV-2 infection, and the mechanisms involved, we used polarized HBE cells differentiated by culture at ALI, BEAS-2B cells and VERO cells, all of which support the replication of SARS-CoV-2 (33–35). We found that culture under hypercapnic conditions (15% CO_2_, PCO_2_ 108) for 2 to 4 days, as compared to culture in normocapnia (5% CO_2_, PCO_2_ 36), increased ACE2 protein expression in HBE (**Figure 1B**), BEAS-2B (**Figure 1C**) and VERO cells (**Figures 1D, E**). Of note, ACE2 mRNA expression was increased after 1 day of culture in hypercapnia in BEAS-2B cells (data not shown).

### 3.2 Hypercapnia increases pseudo-SARS-CoV-2 entry into epithelial cells

**Figure 2:**
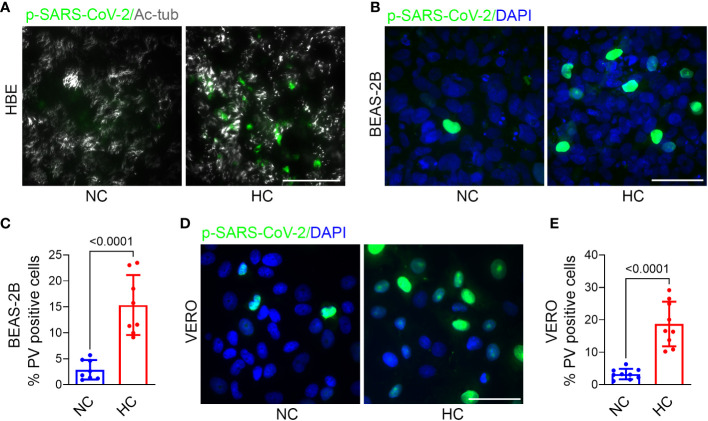
Hypercapnia increases Pseudo-SARS-CoV-2 entry into epithelial cells. HBE **(A)**, BEAS-2B **(B)**, and VERO **(D)** cells were pre-exposed to NC or HC for 2 days, pseudo-SARS-CoV-2 virus (p-SARS-CoV-2, PV) was added, cells were cultured for an additional day in NC or HC, respectively, the fixed. Entry of PV into cells was assessed by IF (representative images in **A**, **B**, **D**) and quantified as the percentage of PV-positive cells in BEAS-2B **(C)** and VERO **(E)** cultures. In **(A)**, ciliated cells were labeled with acetylated-tubulin (Ac-tub). Nuclei were labeled with DAPI. Scale bars = 50 µM. All data are means ± SD. P values for comparisons between groups are shown **(C, E)**.

To determine the effect of hypercapnia on SARS-CoV-2 pseudovirus infection, epithelial cells were cultured in normocapnia or hypercapnia for 2 days, incubated with p-SARS-CoV-2 in normocapnia or hypercapnia for an additional day, then fixed and assessed for GFP expression, i.e. PV positivity, by fluorescence microscopy. We found that culture in elevated CO_2_ increased the percentage of PV positive cells in HBE (**Figure 2A**), BEAS-2B (**Figures 2B, C**), and VERO (**Figures 2D, E**) cell cultures, indicating that hypercapnia increases p-SARS-CoV-2 entry into epithelial cells. Culture of BEAS-2B and VERO cells in hypercapnia after infection with p-SARS-CoV-2 under normocapnic conditions did not increase the percentage of GFP positive cells (**Supplementary Figure 2**), excluding the possibility that hypercapnia increases the apparent percentage of PV positive cells by increasing GFP transcription, translation or fluorescence intensity after viral uptake. The increase in viral entry occurred in the same time frame as the hypercapnia-induced increase in ACE2 protein expression in these cells (**Figures 1A–E**). Of note, when epithelial cells cultured in hypercapnia for 2 days were returned to culture in normocapnia for an additional day, ACE2 protein expression (**Supplementary Figure 3A**) and p-SARS-CoV-2 entry (**Supplementary Figures 3B, C**) were reduced in comparison with cells continuously cultured in elevated CO_2_. Thus, the increases in ACE2 expression and viral entry caused by hypercapnia are reversible.

### 3.3 Hypercapnia increases expression and activation of SREBP2, increases expression of HMGCS1, decreases expression of cholesterol efflux transporter ABCA1, and increases cholesterol accumulation in epithelial cells

**Figure 3:**
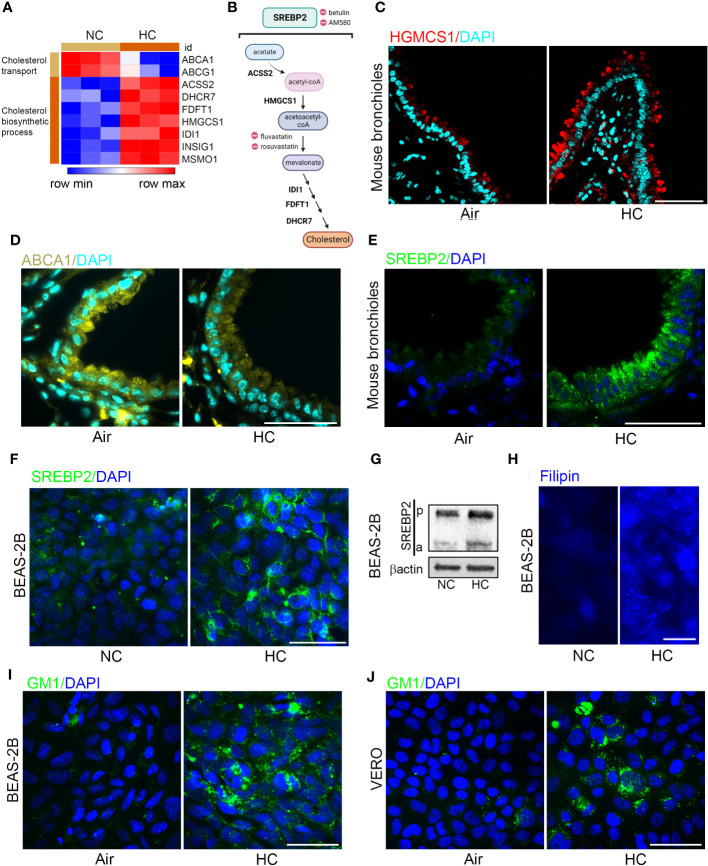
Hypercapnia decreases cholesterol efflux transporters and increases expression and activation of SREBP2, expression of HGMCS1, and cholesterol accumulation in epithelial cells. HBE cells were exposed to NC or HC for 24 h, after which global gene expression was analyzed using Affymetrix GeneChip Hybridization. Heat map shows k-means clustering of cholesterol gene expression profiles, each column represents one sample, and each row represents one transcript **(A)**. Diagram of the major metabolic intermediates in the pathways for synthesis of cholesterol regulated by the transcription factor SREBP2, created with BioRender.com **(B)**. HGMCS1 **(C)**, ABCA1 **(D)**, and SREBP2 **(E)** protein expression was assessed by IF in the bronchi of mice exposed to air or normoxic HC for 7 days. SREBP2 protein expression was assessed by IF **(F)**, and cleavage of pre-SREBP2 (p) to its active form (a) was assessed by immunoblot **(G)** in BEAS-2B cells cultured in NC or HC for 3 days. Cholesterol accumulation was assessed by labeling cholesterol with filipin in BEAS-2B cells **(H)** and with GM1 specifically in lipid rafts in BEAS-2B **(I)** and VERO **(J)** cells after culture in NC or HC for 3 days. Nuclei were stained with DAPI. β-actin was used as loading control in immunoblots. Scale bars = 50 µM, except in **(H)** = 20 µM. .

In a previous transcriptional profiling study (18) we found that culture in hypercapnia increased expression of cholesterol biosynthesis genes including HMGCS1 and decreased expression of ABC transporters which promote cholesterol efflux (**Figures 3A**). These results suggested that culture in hypercapnia might increase cellular cholesterol by upregulating the pathway leading to its synthesis (**Figure 3B**) and decreasing export of cholesterol from the cell. Interestingly, high cellular cholesterol content has been shown to increase SARS-CoV-2 infection (20, 22, 36). Here we found that exposure of mice to normoxic hypercapnia for 7 d increased protein expression of HMGCS1 (**Figure 3C**) and decreased expression of ABCA1 protein expression in bronchial epithelium (**Figure 3D**). Simultaneously, hypercapnia increased protein expression of SREBP2, the master transcription factor that upregulates transcription of cholesterol biosynthesis genes and downregulates transcription of ABC transporters (19), both in mouse bronchial epithelium (**Figure 3E**) and in cultured BEAS-2B cells (**Figures 3F, G**). Furthermore, hypercapnia activated SREBP2, indicated by the increase in expression of its cleaved isoform (a) (**Figure 3G**), which is released from the endoplasmic reticulum and translocated to the nucleus. In sum, these effects of hypercapnia would be expected to increase cholesterol accumulation in epithelial cells. Indeed, culture in hypercapnia increased overall cellular cholesterol in BEAS-2B cells (**Figure 3H**) and cholesterol localization specifically in lipid rafts in BEAS-2B and VERO cells (**Figures 3I, J**).

### 3.4 Hypercapnia increases ACE2 protein expression, p-SARS-CoV-2 entry into cells, and cholesterol accumulation independently of extracellular acidosis

Since increasing CO_2_ exposure lowers the pH of the culture media (37), we assessed whether the increases in ACE2 expression, p-SARS-CoV-2 entry, and cellular cholesterol accumulation could be due to extracellular acidosis, as opposed to the increase in CO_2_, per se. In these studies, we buffered culture media with Tris-HCl and Tris base to reach pH 7.4 with 15% CO_2_, and pH 7.2 with 5% CO_2_ (non–Tris-buffered media in 5 and 15% CO_2_, have pH 7.2 and pH 7.4, respectively). When epithelial cells were cultured in these media, we observed greater ACE2 protein expression (**Supplementary Figures 4A, B**) and a higher percentage of cells staining positively for p-SARS-CoV-2 (**Supplementary Figures 4C, D**) in hypercapnia (15% CO_2_) than in normocapnia (5% CO_2_), regardless of whether the pH was 7.2 or 7.4. Likewise, hypercapnia increased cholesterol accumulation (**Supplementary Figures 4E–G**) at both pH 7.2 and pH 7.4. Thus, the effects of hypercapnia on ACE2 expression, p-SARS-CoV-2 entry and cholesterol accumulation in epithelial cells are not attributable to extracellular acidosis, but instead result from the higher PCO_2_, independent of pH.

### 3.5 Inhibition of cholesterol synthesis and cholesterol depletion both block the CO_2_-induced increase in ACE2 and p-SARS-CoV-2 entry into epithelial cells

**Figure 4:**
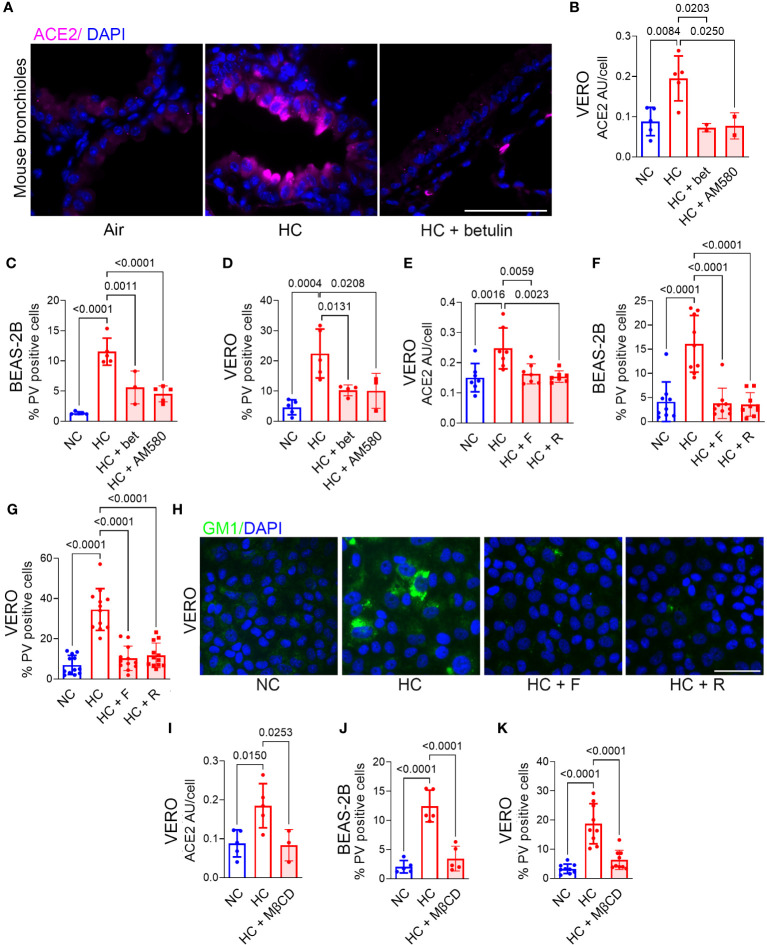
Inhibition of cholesterol synthesis or depletion of cholesterol blocks the hypercapnia-induced increases in ACE2 and Pseudo-SARS-CoV-2 entry into epithelial cells. Mice were exposed to normoxic HC for 7 days, or room air as control. Betulin (5 mg/kg body weight) or vehicle were administrated 3 days before and at the start of HC exposure, and ACE2 protein expression in mouse bronchi was assessed by IF **(A)**. BEAS-2B and VERO cells were treated with the SREBP2 inhibitors betulin (bet, 7.5 µM) or AM580 (20 µM), the statins fluvastatin (F, 50 nM) or rosuvastatin (R, 0.5 µM), or the cholesterol depleting agent methyl-β-cycle odextrin (MβCD, 100 µM) and incubated in NC or HC for 2 days. Pseudo-SARS-CoV-2 virus (PV) or vehicle were then added, and cells were cultured for an additional day in NC or HC, respectively, then fixed and stained. ACE2 expression was quantified as relative fluorescence intensity per cell, expressed in arbitrary units (AU) **(B, E, I)**. Viral entry was quantified as the percentage of PV-positive cells in BEAS-2B **(C, F, J)** and VERO **(D, G, K)** cultures. Nuclei were stained with DAPI. All data are means ± SD. P values for comparisons between groups are shown.

Hypercapnia increases ACE2 expression (**Figures 1A–E**), p-SARS-CoV-2 internalization (**Figures 2A–E**) and cellular cholesterol (**Figures 3H–J**) in epithelial cells. Thus, we investigated the impact of blocking cholesterol synthesis on ACE2 protein and virus entry. Betulin, a plant-derived pentacyclic triterpene (38) that inhibits activation of SREBP2, thereby blocking its ability to regulate cholesterol synthesis and efflux genes (39), prevented the hypercapnia-induced increase in ACE2 expression in mouse bronchial epithelium *in vivo* (**Figure 4A**) and VERO cells *in vitro* (**Figure 4B**). AM580, a synthetic retinoid derivative that inhibits SREBP1/2 binding to promoter/enhancer regions of multiple lipogenic genes (40), similarly blocked the effect of hypercapnia in VERO cells (**Figure 4B**). In addition, both betulin and AM580 blocked p-SARS-CoV-2 entry in BEAS-B (**Figure 4C**) and VERO (**Figure 4D**) cells. Betulin and AM580 are not approved for use in humans, while statins are widely used lipid-lowering agents in the clinic. Statins inhibit 3-hydroxy-3-methyl glutaryl (HMG)-CoA reductase (HMGCR), a rate-limiting enzyme that catalyzes the conversion of HMG-CoA into L-mevalonate (**Figure 3B**), thereby inhibiting cholesterol biosynthesis (41, 42). Given their safety record, efficacy and affordability, statins are attractive candidates for host-directed therapy against infectious diseases (43). Moreover, several studies suggest a significant protective effect of statins on patients with COVID-19 (23–26). Therefore, we tested fluvastatin and rosuvastatin, and found that both statins block ACE2 expression in VERO cells (**Figure 4E**) and p-SARS-CoV-2 entry in BEAS-2B and VERO cells (**Figures 4F, G**). Of note, none of the inhibitors used in the present work reduced p-SARS-CoV-2 entry under normocapnic culture conditions (**Supplementary Figure 5**), suggesting that this effect occurs only when cholesterol accumulation is increased and not in the basal state. Fluvastatin and rosuvastatin also inhibited the hypercapnia-induced increase in cholesterol in lipid rafts in VERO (**Figure 4H**) and in BEAS-2B (**Supplementary Figure 6A**) cells. Of note, uptake of statins into cells is mediated by organic anion transporting polypeptide (OATP)1B1 (encoded by *SLCO1B1*), while efflux of statins from cells is mediated by the breast cancer resistance protein (BCRP, encoded by *ABCG2*) (44). In previously published work, we confirmed that *SLCO1B1* and *ABCG2* are transcribed in HBE cells (18). We show here that culture in hypercapnia did not alter mRNA expression of either of these statin transport genes (**Supplementary Figure 7**), excluding the possibility that statins selectively block hypercapnia-induced increases in cholesterol and ACE2 expression by altering drug transport into or out of epithelial cells. Cholesterol depletion from lipid rafts using methyl-β-cyclodextrin (MβCD) (45) decreases the interaction between the coronavirus spike protein and the ACE2 receptor (20, 46). Thus, we assessed the impact of MβCD on the effect of elevated CO_2_ in VERO and BEAS-2B cells, and found that it blocked the hypercapnia-induced increases in ACE2 expression (**Figure 4I**) and p-SARS-CoV-2 entry (**Figures 4J, K**). These results indicate that blocking cholesterol synthesis and increasing its removal from the cell both prevent the increases in ACE2 protein expression and viral entry caused by elevated CO_2_.

### 3.6 Cigarette smoke extract increases epithelial cell ACE2 expression, p-SARS-CoV-2 entry, and cholesterol accumulation in a manner not additive to the effects of hypercapnia

**Figure 5:**
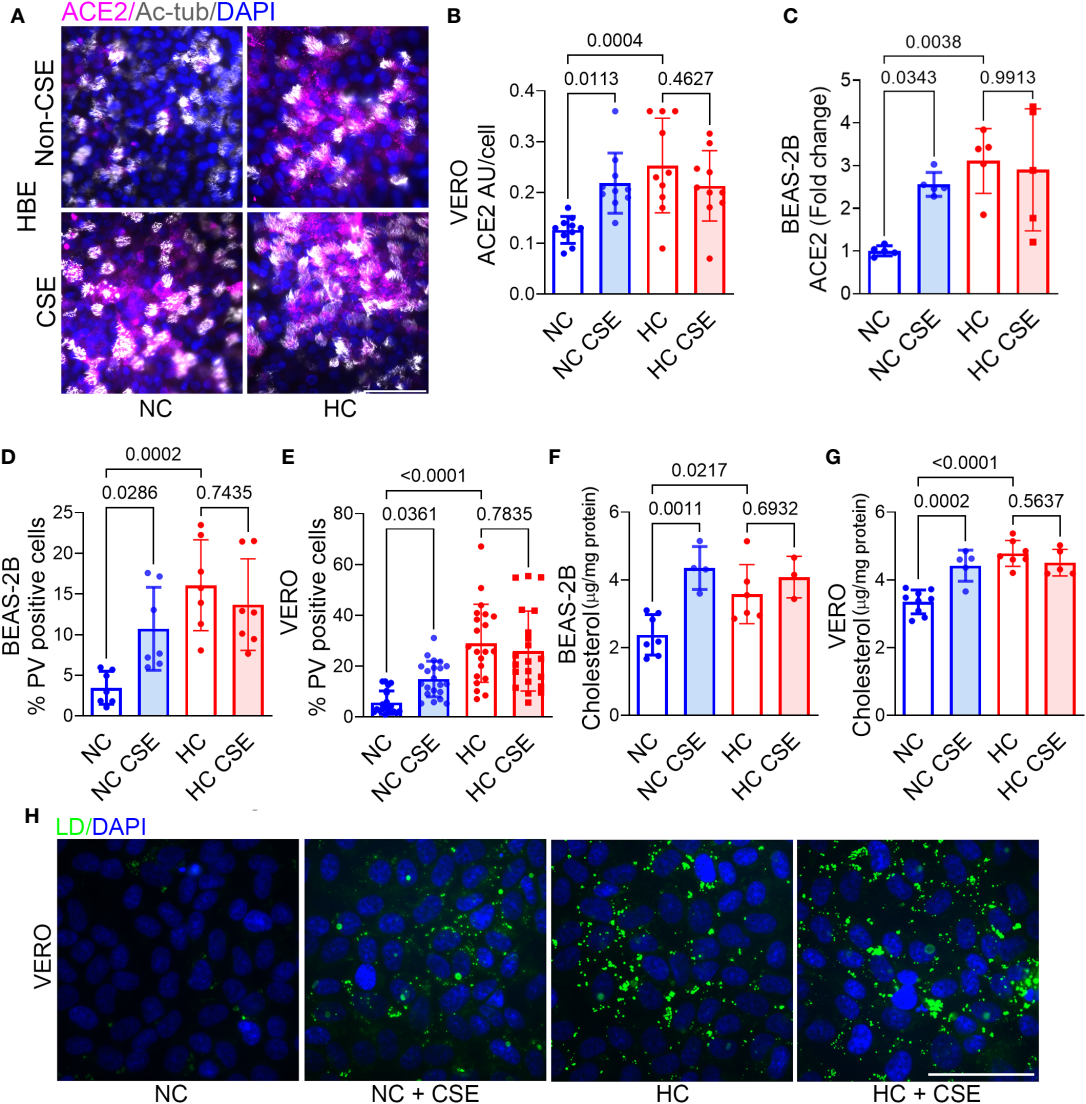
Cigarette smoke extract (CSE) increases ACE2 expression, Pseudo-SARS-CoV-2 entry, and cholesterol accumulation in epithelial cells in a manner not additive to the effects of hypercapnia. HBE, BEAS-2B and VERO cells were exposed to cigarette smoke CSE, (1 μg/ml) during culture in NC or HC for 2 days, pseudo-SARS-CoV-2 virus (PV) or vehicle were added, and cells were cultured for an additional day in NC or HC, respectively, then fixed and stained. ACE2 protein expression was assessed by IF in HBE **(A)** and VERO cells **(B)** and quantified in the latter as relative fluorescence intensity per cells, expressed in arbitrary units (AU) **(B)**. ACE2 mRNA expression was assessed by qPCR after exposure of BEAS-2B cells to CSE in NC or HC for 1 day **(C)**. Viral entry was assessed by IF and quantified as the percentage of PV-positive cells in BEAS-2B **(D)** and VERO **(E)** cultures. Cholesterol accumulation was assessed by Amplex red assay in BEAS-2B **(F)** and VERO **(G)** cell lysates, and lipid droplets (LD) in VERO cells were labeled with LipidSpot 488 **(H)**. Ciliated cells were labeled with acetylated-tubulin (Ac-tub) **(A)**. Nuclei were labeled with DAPI. Scale bars = 50 µM. All data are means ± SD. P values for comparisons between groups are shown.

Active tobacco smoking is associated with increased mortality in patients with chronic hypercapnia (47) and COVID-19 (48). In addition, exposure to cigarette smoke increases expression of ACE2 in the respiratory tract (49), promotes cholesterol accumulation and decreases expression of ABC transporters in human bronchial epithelial cells (50). Since patients with advanced lung disease and hypercapnia may continue to smoke, we examined the impact of exposure to CSE, alone and in combination with hypercapnia, on ACE2 expression, p-SARS-CoV-2 entry, and cholesterol accumulation in epithelial cells. We found that CSE increases ACE2 protein at day 3 (**Figures 5A, B**) and mRNA expression at day 1 (**Figure 5C**), p-SARS-CoV-2 entry (**Figures 5D, E**) into epithelial cells in conjunction with increased cholesterol accumulation (**Figures 5F–H**). The effects of CSE and hypercapnia were similar in magnitude, but they were not additive (**Figures 5A–H**). These results suggest that hypercapnia and cigarette smoking independently augment cholesterol accumulation by similar mechanisms, both increasing ACE2 expression and p-SARS-CoV-2 entry, but in a non-additive, non-synergistic manner.

### 3.7 Statins block CSE-induced increases in lipid raft cholesterol, ACE2 protein expression and p-SARS-CoV-2 entry into epithelial cells

**Figure 6:**
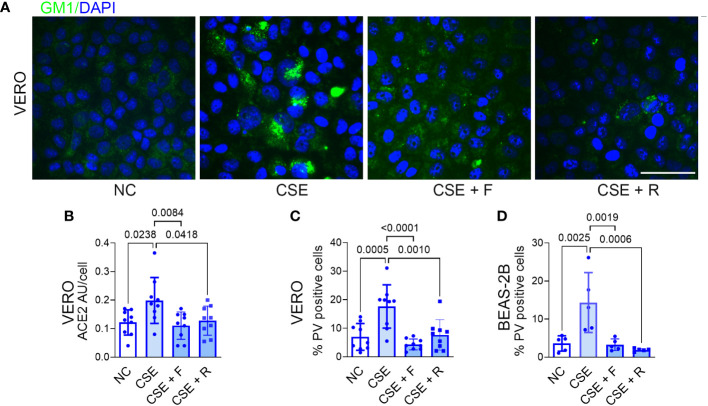
Statins block CSE-induced increases in lipid raft cholesterol, ACE2 protein expression and Pseudo-SARS-CoV-2 entry into epithelial cells. VERO and BEAS-2B cells were treated with fluvastatin (F, 50 nM) or rosuvastatin (R, 0.5 µM) for 1 day prior to and during exposure to CSE (1 µg/ml) for 2 days, pseudo-SARS-CoV-2 virus (PV) or vehicle were added, and cells were cultured for an additional day, in NC throughout, then fixed and stained. Cholesterol accumulation in lipid rats was assessed by labeling GM1 **(A)**. ACE2 protein was assessed by IF and quantified as relative fluorescence intensity per cell, expressed in arbitrary units (AU) **(B)**. Viral entry was assessed by IF and quantified as the percentage of PV-positive cells in VERO **(C)** and BEAS-2B cells **(D)**. Nuclei were labeled DAPI. Scale bars = 50 µM. All data are means ± SD. P values for comparisons between groups are shown.

As noted above, fluvastatin and rosuvastatin blocked hypercapnia-induced increases in cholesterol accumulation, ACE2 protein expression, and p-SARS-CoV-2 entry into epithelial cells (**Figures 4E–H**). Both statins also blocked the increase in cholesterol in lipid rafts stimulated by CSE in VERO (**Figure 6A**) and BEAS-2B cells (**Supplementary Figure 6B**). Likewise, the statins blocked the CSE-induced increases in ACE2 expression (**Figure 6B**) and p-SARS-CoV-2 internalization (**Figures 6C, D**). Thus, inhibiting cholesterol synthesis with statins blocks cellular cholesterol accumulation caused by cigarette smoke, resulting in decreased expression of ACE2 and reduced entry of p-SARS-CoV-2 into epithelial cells.

## 4 Discussion

In the present work we show that hypercapnia increases ACE2 expression and p-SARS-CoV-2 entry in bronchial epithelial cells. Hypercapnia also increases total cellular cholesterol and cholesterol in epithelial cell lipid rafts and lipid droplets. The CO_2_-induced increase in cholesterol was associated with increased expression of SREBP2, and a corresponding increase in expression of the cholesterol synthesis enzymes, which are activated by SREBP2, and decrease in expression of the cholesterol efflux transporter ABCA1, which is repressed by SREBP2. Moreover, inhibiting cholesterol synthesis (by inhibiting SREBP2 or HMGCR) or depleting cholesterol from lipid rafts each blocked the increase in ACE2 and p-SARS-CoV-2 entry in cells exposed to elevated CO_2_. These findings establish a causal role for the increase in cholesterol in driving the hypercapnia-induced increases in ACE2 expression and viral uptake. We also found that CSE increased ACE2 expression and p-SARS-CoV-2 internalization by increasing lipid raft cholesterol, and that inhibiting cholesterol synthesis blocked the CSE effect. Thus, hypercapnia and CSE act through a common cholesterol-dependent mechanism to upregulate ACE2 and enhance p-SARS-CoV-2 entry into respiratory epithelial cells.

SARS-CoV-2 relies on its obligate receptor ACE2 to bind and enter host cells (51, 52). We found that hypercapnia increases ACE2 expression in bronchial epithelium *in vivo* and *in vitro*. Several studies show that ACE2 expression is increased in bronchial epithelium of patients with pre-existing conditions associated with severe COVID-19 (6–9) that often are accompanied by elevated CO_2_ (53–55). Moreover, these comorbidities and/or hypercapnia increase the risk of mortality in patients with COVID-19 (1, 3, 4, 15) and other respiratory infections (10, 14, 56, 57). These findings suggest that hypercapnia may contribute to the upregulated ACE2 expression in patients with pre-existing conditions, increasing the probability of SARS-CoV-2 infection and of severe COVID-19. Hypercapnia upregulates ACE2 expression, thus available binding sites for p-SARS-CoV-2 are increased, concurring with increased virus uptake in bronchial epithelial cells exposed to high CO_2_. Single-cell RNA-sequencing data show that the level of ACE2 expression in various organs correlates with the potential for infection by SARS-CoV-2 (58). Also, among patients infected with SARS-CoV-2, transmembrane ACE2 transcript levels correlated with SARS-CoV-2 viral load in a nasopharyngeal sampling study (59), highlighting the importance of ACE2 expression in viral infection and replication. Notably, the effect of hypercapnia on ACE2 expression and p-SARS-CoV2 uptake were reversed when cells were returned to culture in normocapnia. This finding is similar to our previous observations that that hypercapnic inhibition of IL-6 expression (37) and autophagy (60) and the hypercapnia-induced increases in mortality of bacterial (27) and influenza A (16) pneumonia in mice were reversible, as well. The reversibility of hypercapnia-induced defects in antiviral and antibacterial host defense may in part explain why use of noninvasive ventilation to reduce arterial PCO_2_ prolonged the time to hospital readmission and decreased mortality in patients with severe COPD and chronic hypercapnic respiratory failure (61–63).

We also show that hypercapnia increases total cellular cholesterol and cholesterol in lipid rafts and lipid droplets. Various coronaviruses interact with a diverse repertoire of receptors located on lipid rafts, which provide a platform that concentrates receptors mediating entry of viruses and other ligands into cells. By binding viral spike protein, ACE2 localized to lipid rafts is fundamental to the initial step of SARS-CoV-2 infection (20, 64, 65), as is also the case for SARS-CoV, which also uses ACE2 as its cellular receptor (20). Notably, increased uptake of cholesterol into cell membranes in obese and diabetic mice (also comorbidities for COVID-19 in humans) causes ACE2 to move to endocytic ganglioside GM1 in lipid rafts, which optimally localizes bound virus for entry into the cell (21). MβCD disrupts lipid rafts and the association of ACE2 with GM1 lipids, thus decreasing viral endocytosis, and thereby infectivity (46, 65). In the present report, we found that MβCD also inhibited the hypercapnia-induced increases in ACE2 and p-SARS-CoV-2 internalization. These data highlight the importance of cholesterol-rich lipid rafts for efficient interaction between the viral surface protein and the cellular receptor in bronchial epithelium. The hypercapnia-induced increase in lipid droplet cholesterol is also important, since lipid droplets accumulate in cells infected with SARS-CoV-2, both *in vitro* and in the human lung (66). Moreover, lipid droplets (LD) were found in close apposition with SARS-CoV-2 proteins and double-stranded (ds)-RNA in infected VERO cells, suggesting that lipid droplets serve as a platform for viral assembly (67). In these cells, pharmacological modulation of lipid droplet formation inhibited SARS-CoV-2 replication and also reduced synthesis of pro-inflammatory mediators (67). Together with our findings, these observations suggest that in addition to enhancing viral entry by increasing cholesterol in lipid rafts, elevated CO_2_ may also enhance virus assembly by increasing cholesterol in lipid droplets, thereby increasing SARS-CoV-2 infectivity and spread within the lung.

Of note, we found that hypercapnia-induced increases in epithelial cell ACE2 expression, p-SARS-CoV-2 entry and cellular cholesterol were not due to extracellular acidosis. Similarly, we previously showed that hypercapnia suppressed cytokine expression, autophagy, and antiviral genes in cultured macrophages, and increased the mortality of bacterial pneumonia and influenza A infection in mice, all in a pH-independent manner (16, 37, 60). In other studies, hypercapnia downregulated alveolar epithelial Na,K-ATPase activity and inhibited alveolar fluid clearance in the rat lung, triggered internalization of Na,K-ATPase by activating AMP-activated kinase and PKC-zeta, and inhibited lung fibroblasts and alveolar epithelial cells proliferation independently of pH (68–70). Taken together, these observations suggest that the effects of hypercapnia on lung epithelial cells and macrophages are mediated by pathway(s) in which increases in molecular CO_2_ are sensed and trigger specific signaling events within cells.

We used two approaches to show that inhibition of cholesterol synthesis blocked the hypercapnia-induced increases in ACE2 expression and p-SARS-CoV-2 entry into cells. First, we inhibited SREBP2, the master regulator of cholesterol synthesis (19, 71), with betulin (39) or AM580 (40), and second, we inhibited HMGCR (42), a rate-controlling enzyme of the cholesterol biosynthetic pathway with fluvastatin or rosuvastatin (See **Figure 1B**). SREBP2 inhibition reduced expression cholesterol synthesis genes (40), which we reported previously were upregulated by elevated CO_2_ (18). Interestingly, SREBP2 is activated and may play a role in cytokine storm in patients with COVID-19 (72). Betulin and its derivative betulinic acid have antiviral activity against influenza A virus (73), HIV-1 (74), Echo-6 virus (75), MERS-CoV (40), and AM580 blocks MERS-CoV, SARS-CoV, Zika, H1N1 and EV-A71 virus replication *in vitro* (40). However, betulin and AM580 have not been tested for their antiviral activity clinically, and neither is available for use in humans. On the other hand, statins, which have been in clinical use as lipid-lowering agents for decades, are attractive candidates for host-directed therapy against viral infections (43). While the data are not entirely consistent, several studies suggest that chronic statin therapy may be protective against severe disease in patients with COVID-19 (23–26). In the present work we show that statins block hypercapnia-induced increases in ACE2 expression and p-SARS-CoV-2 entry. Of note, statins and the other cholesterol inhibitors did not block virus entry under normocapnic conditions, suggesting that these drugs may be most effective in situations where cellular cholesterol is increased, such in patients with hypercapnia, and those who smoke cigarettes. Besides lowering cholesterol, statins may also provide benefit through their anti-inflammatory actions (23) and their ability to downregulate proteins that modulate protein translation and viral replication (76).

Active smoking is also associated with increased mortality in patients with chronic hypercapnia (47) and COVID-19 (1, 48, 77). Similar to hypercapnia, CSE increased cellular cholesterol, ACE2 expression and p-SARS-CoV-2 entry into epithelial cells. Notably, the effects of hypercapnia and CSE were not additive, suggesting that each may maximally increase the capacity of epithelial cells to increase their cholesterol content. The complexity of cigarette smoke suggests that it may increase cellular cholesterol and ACE2 expression by multiple mechanisms. One likely mechanism underlying the increase in cholesterol is oxidative stress-induced activation of SREBP2 (78) resulting in increased expression of cholesterol synthesis enzymes and decreased expression of cholesterol efflux transporters (79) (**Figure 3A**). ACE2 expression is also increased by cigarette smoked-induced oxidative stress via HIF-1α-dependent (80) and HIF-1α-independent (81) mechanisms, and by nicotine acting via the α7-nAChR nicotinic receptor (82).

In the current study, we found that hypercapnia decreased expression of the cholesterol efflux transporter ABCA1 in mouse airway epithelium *in vivo*, similar to our previous observation that elevated CO_2_ downregulated transcription of ABC transporters in bronchial epithelial cells *in vitro* (18). CSE has also been shown to decreases expression of ABC transporters in human bronchial epithelial cells (50). A major function of these transporters is to regulate cholesterol content in lipid rafts and promote the efflux of intracellular free cholesterol across the plasma membrane to the extracellular space, where it is bound by apolipoproteins in the circulation (83). Thus, in addition to increasing cholesterol synthesis, hypercapnia and CSE both increase cholesterol accumulation in the cell by downregulating ABC-mediated cholesterol efflux.

In summary, in the present work we show that hypercapnia and cigarette smoke extract increase ACE2 expression and p-SARS-CoV-2 entry in epithelial cells by increasing cholesterol in lipid rafts, which provide a platform for localization of ACE2 and a portal for entry of virus into the cell. These results suggest that pharmacologic interventions to decrease cellular cholesterol may reduce susceptibility to SARS-CoV-2 infection and/or the severity of COVID-19 disease in patients with hypercapnia and in cigarette smokers. In addition, the observation that the increase in epithelial ACE2 expression caused by elevated CO_2_ is reversible suggests that ventilatory support strategies to reduce hypercapnia might also ameliorate SARS-CoV-2 infection and adverse outcomes of COVID-19 in patients with acute or chronic hypercapnic respiratory failure.

## Data availability statement

Publicly available datasets were analyzed in this study. This data can be found here: GEO GSE110362.

## Ethics statement

Ethical approval was not required for the studies on humans in accordance with the local legislation and institutional requirements because only commercially available established cell lines were used. The animal study was approved by Institutional Animal Care and Use Committee of Northwestern University. The study was conducted in accordance with the local legislation and institutional requirements.

## Author contributions

PS and SC-M conceived and designed the experiments. FC, AM, and SC-M. performed the experiments. AM, PS, and SC-M analysed and interpreted the data. GB contributed reagents or analytic tools, PS and SC-M wrote the original draft. All authors contributed to the article and approved the submitted version.

## References

[1] WHO. WHO coronavirus (COVID-19) dashboard. Available at: https://covid19.who.int/.

[2] CDC. COVID-19 people with certain medical conditions (2023). Available at: https://www.cdc.gov/coronavirus/2019-ncov/need-extra-precautions/people-with-medical-conditions.html.

[3] Bucholc M Bradley D Bennett D Patterson L Spiers R Gibson D. Identifying pre-existing conditions and multimorbidity patterns associated with in-hospital mortality in patients with COVID-19. Sci Rep (2022) 12(1):17313. doi: 10.1038/s41598-022-20176-w 36243878 PMC9568958

[4] Gerayeli FV Milne S Cheung C Li X Yang CWT Tam A. COPD and the risk of poor outcomes in COVID-19: A systematic review and meta-analysis. EClinicalMedicine (2021) 33:100789. doi: 10.1016/j.eclinm.2021.100789 33758801 PMC7971471

[5] Yang J Petitjean SJL Koehler M Zhang Q Dumitru AC Chen W. Molecular interaction and inhibition of SARS-CoV-2 binding to the ACE2 receptor. Nat Commun (2020) 11(1):4541. doi: 10.1038/s41467-020-18319-6 32917884 PMC7486399

[6] Pinto BGG Oliveira AER Singh Y Jimenez L Goncalves ANA Ogava RLT. ACE2 expression is increased in the lungs of patients with comorbidities associated with severe COVID-19. J Infect Dis (2020) 222(4):556–63. doi: 10.1093/infdis/jiaa332 PMC7377288 32526012

[7] Jacobs M Van Eeckhoutte HP Wijnant SRA Janssens W Joos GF Brusselle GG. Increased expression of ACE2, the SARS-CoV-2 entry receptor, in alveolar and bronchial epithelium of smokers and COPD subjects. Eur Respir J (2020) 56(2):2002378. doi: 10.1183/13993003.02378-2020 32675207 PMC7366177

[8] Higham A Singh D. Increased ACE2 expression in bronchial epithelium of COPD patients who are overweight. Obes (Silver Spring) (2020) 28(9):1586–9. doi: 10.1002/oby.22907 PMC7276828 32428380

[9] Zamorano Cuervo N Grandvaux N. ACE2: Evidence of role as entry receptor for SARS-CoV-2 and implications in comorbidities. Elife (2020) 9:e61390. doi: 10.7554/eLife.61390 33164751 PMC7652413

[10] Belkin RA Henig NR Singer LG Chaparro C Rubenstein RC Xie SX. Risk factors for death of patients with cystic fibrosis awaiting lung transplantation. Am J Respir Crit Care Med (2006) 173(6):659–66. doi: 10.1164/rccm.200410-1369OC PMC2662949 16387803

[11] De Serres G Lampron N La Forge J Rouleau I Bourbeau J Weiss K. Importance of viral and bacterial infections in chronic obstructive pulmonary disease exacerbations. J Clin Virol (2009) 46(2):129–33. doi: 10.1016/j.jcv.2009.07.010 PMC7108387 19665425

[12] Murtagh P Giubergia V Viale D Bauer G Pena HG. Lower respiratory infections by adenovirus in children. Clinical features and risk factors for bronchiolitis obliterans and mortality. Pediatr Pulmonology (2009) 44(5):450–6. doi: 10.1002/ppul.20984 19360848

[13] Nin N Muriel A Penuelas O Brochard L Lorente JA Ferguson ND. Severe hypercapnia and outcome of mechanically ventilated patients with moderate or severe acute respiratory distress syndrome. Intensive Care Med (2017) 43(2):200–8. doi: 10.1007/s00134-016-4611-1 PMC5630225 28108768

[14] Sin DD Man SF Marrie TJ. Arterial carbon dioxide tension on admission as a marker of in-hospital mortality in community-acquired pneumonia. Am J Med (2005) 118(2):145–50. doi: 10.1016/j.amjmed.2004.10.014 15694899

[15] Tsonas AM Botta M Horn J Morales-Quinteros L Artigas A Schultz MJ. Clinical characteristics, physiological features, and outcomes associated with hypercapnia in patients with acute hypoxemic respiratory failure due to COVID-19—insights from the PRoVENT-COVID study. J Crit Care (2022) 69:154022. doi: 10.1016/j.jcrc.2022.154022 35339900 PMC8947815

[16] Casalino-Matsuda SM Chen F Gonzalez-Gonzalez FJ Nair A Dib S Yemelyanov A. Hypercapnia suppresses macrophage antiviral activity and increases mortality of influenza A infection via Akt1. J Immunol (2020) 205(2):489–501. doi: 10.4049/jimmunol.2000085 32540997 PMC7343622

[17] Casalino-Matsuda SM Chen F Gonzalez-Gonzalez FJ Matsuda H Nair A Abdala-Valencia H. Myeloid zfhx3 deficiency protects against hypercapnia-induced suppression of host defense against influenza A virus. bioRxiv (2023). doi: 10.1101/2023.02.28.530480 PMC11143927 38227369

[18] Casalino-Matsuda SM Wang N Ruhoff PT Matsuda H Nlend MC Nair A. Hypercapnia alters expression of immune response, nucleosome assembly and lipid metabolism genes in differentiated human bronchial epithelial cells. Sci Rep (2018) 8(1):13508. doi: 10.1038/s41598-018-32008-x 30202079 PMC6131151

[19] Horton JD Goldstein JL Brown MS. SREBPs: activators of the complete program of cholesterol and fatty acid synthesis in the liver. J Clin Invest (2002) 109(9):1125–31. doi: 10.1172/JCI0215593 PMC150968 11994399

[20] Palacios-Rapalo SN De Jesus-Gonzalez LA Cordero-Rivera CD Farfan-Morales CN Osuna-Ramos JF Martinez-Mier G. Cholesterol-rich lipid rafts as platforms for SARS-CoV-2 entry. Front Immunol (2021) 12:796855. doi: 10.3389/fimmu.2021.796855 34975904 PMC8719300

[21] Wang H Yuan Z Pavel MA Jablonski SM Jablonski J Hobson R. The role of high cholesterol in SARS-CoV-2 infectivity. J Biol Chem (2023) 299(6):104763. doi: 10.1016/j.jbc.2023.104763 37119851 PMC10140059

[22] Dai J Wang H Liao Y Tan L Sun Y Song C. Coronavirus infection and cholesterol metabolism. Front Immunol (2022) 13:791267. doi: 10.3389/fimmu.2022.791267 35529872 PMC9069556

[23] Kouhpeikar H Khosaravizade Tabasi H Khazir Z Naghipour A Mohammadi Moghadam H Forouzanfar H. Statin use in COVID-19 hospitalized patients and outcomes: A retrospective study. Front Cardiovasc Med (2022) 9:820260. doi: 10.3389/fcvm.2022.820260 35282379 PMC8907562

[24] Santosa A Franzen S Natman J Wettermark B Parmryd I Nyberg F. Protective effects of statins on COVID-19 risk, severity and fatal outcome: a nationwide Swedish cohort study. Sci Rep (2022) 12(1):12047. doi: 10.1038/s41598-022-16357-2 35835835 PMC9282150

[25] Wang LK Kuo YF Westra J Raji MA Albayyaa M Allencherril J. Association of cardiovascular medications with adverse outcomes in a matched analysis of a national cohort of patients with COVID-19. Am J Med Open (2023) 9:100040. doi: 10.1016/j.ajmo.2023.100040 37207280 PMC10032048

[26] Bouillon K Baricault B Semenzato L Botton J Bertrand M Drouin J. Association of statins for primary prevention of cardiovascular diseases with hospitalization for COVID-19: A nationwide matched population-based cohort study. J Am Heart Assoc (2022) 11(12):e023357. doi: 10.1161/JAHA.121.023357 35699173 PMC9238639

[27] Gates KL Howell HA Nair A Vohwinkel CU Welch LC Beitel GJ. Hypercapnia impairs lung neutrophil function and increases mortality in murine pseudomonas pneumonia. Am J Respir Cell Mol Biol (2013) 49(5):821–8. doi: 10.1165/rcmb.2012-0487OC PMC3931098 23777386

[28] Casalino-Matsuda SM Monzon ME Forteza RM. Epidermal growth factor receptor activation by epidermal growth factor mediates oxidant-induced goblet cell metaplasia in human airway epithelium. Am J Respir Cell Mol Biol (2006) 34(5):581–91. doi: 10.1165/rcmb.2005-0386OC PMC2644222 16424381

[29] Stirling DR Swain-Bowden MJ Lucas AM Carpenter AE Cimini BA Goodman A. CellProfiler 4: improvements in speed, utility and usability. BMC Bioinf (2021) 22(1):433. doi: 10.1186/s12859-021-04344-9 PMC8431850 34507520

[30] Shigemura M Lecuona E Angulo M Homma T Rodriguez DA Gonzalez-Gonzalez FJ. Hypercapnia increases airway smooth muscle contractility via caspase-7-mediated miR-133a-RhoA signaling. Sci Trans Med (2018) 10(457):eaat1662. doi: 10.1126/scitranslmed.aat1662 PMC6889079 30185650

[31] Dada LA Welch LC Magnani ND Ren Z Han H Brazee PL. Hypercapnia alters stroma-derived Wnt production to limit beta-catenin signaling and proliferation in AT2 cells. JCI Insight (2023) 8(4):e159331. doi: 10.1172/jci.insight.159331 36626234 PMC9977495

[32] Knight AC Montgomery SA Fletcher CA Baxter VK. Mouse models for the study of SARS-CoV-2 infection. Comp Med (2021) 71(5):383–97. doi: 10.30802/AALAS-CM-21-000031 PMC8594264 34610856

[33] Hao S Ning K Kuz CA Vorhies K Yan Z Qiu J. Long-term modeling of SARS-coV-2 infection of *in vitro* cultured polarized human airway epithelium. mBio (2020) 11(6):e02852–20. doi: 10.1128/mBio.02852-20 PMC7649230 33158999

[34] Ogando NS Dalebout TJ Zevenhoven-Dobbe JC Limpens R van der Meer Y Caly L. SARS-coronavirus-2 replication in Vero E6 cells: replication kinetics, rapid adaptation and cytopathology. J Gen Virol (2020) 101(9):925–40. doi: 10.1099/jgv.0.001453 PMC7654748 32568027

[35] Zhou Y Wang M Li Y Wang P Zhao P Yang Z. SARS-CoV-2 Spike protein enhances ACE2 expression via facilitating Interferon effects in bronchial epithelium. Immunol Lett (2021) 237:33–41. doi: 10.1016/j.imlet.2021.06.008 34228987 PMC8254647

[36] Tang Y Hu L Liu Y Zhou B Qin X Ye J. Possible mechanisms of cholesterol elevation aggravating COVID-19. Int J Med Sci (2021) 18(15):3533–43. doi: 10.7150/ijms.62021 PMC8436106 34522180

[37] Wang N Gates KL Trejo H Favoreto S Jr. Schleimer RP Sznajder JI. Elevated CO2 selectively inhibits interleukin-6 and tumor necrosis factor expression and decreases phagocytosis in the macrophage. FASEB J (2010) 24(7):2178–90. doi: 10.1096/fj.09-136895 PMC2887258 20181940

[38] Alakurtti S Makela T Koskimies S Yli-Kauhaluoma J. Pharmacological properties of the ubiquitous natural product betulin. Eur J Pharm Sci (2006) 29(1):1–13. doi: 10.1016/j.ejps.2006.04.006 16716572

[39] Tang JJ Li JG Qi W Qiu WW Li PS Li BL. Inhibition of SREBP by a small molecule, betulin, improves hyperlipidemia and insulin resistance and reduces atherosclerotic plaques. Cell Metab (2011) 13(1):44–56. doi: 10.1016/j.cmet.2010.12.004 21195348

[40] Yuan S Chu H Chan JF Ye ZW Wen L Yan B. SREBP-dependent lipidomic reprogramming as a broad-spectrum antiviral target. Nat Commun (2019) 10(1):120. doi: 10.1038/s41467-018-08015-x 30631056 PMC6328544

[41] Endo A Kuroda M Tsujita Y. ML-236A, ML-236B, and ML-236C, new inhibitors of cholesterogenesis produced by Penicillium citrinium. J Antibiot (Tokyo) (1976) 29(12):1346–8. doi: 10.7164/antibiotics.29.1346 1010803

[42] Istvan ES Deisenhofer J. Structural mechanism for statin inhibition of HMG-CoA reductase. Science (2001) 292(5519):1160–4. doi: 10.1126/science.1059344 11349148

[43] Parihar SP Guler R Brombacher F. Statins: a viable candidate for host-directed therapy against infectious diseases. Nat Rev Immunol (2019) 19(2):104–17. doi: 10.1038/s41577-018-0094-3 30487528

[44] Lonnberg KI Tornio A Hirvensalo P Keskitalo J Mustaniemi AL Kiiski JI. Real-world pharmacogenetics of statin intolerance: effects of SLCO1B1, ABCG2 , and CYP2C9 variants. Pharmacogenet Genomics (2023) 33(7):153–60. doi: 10.1097/FPC.0000000000000504 PMC10399933 37490620

[45] Mahammad S Parmryd I. Cholesterol depletion using methyl-beta-cyclodextrin. Methods Mol Biol (2015) 1232:91–102. doi: 10.1007/978-1-4939-1752-5_8 25331130

[46] Glende J Schwegmann-Wessels C Al-Falah M Pfefferle S Qu X Deng H. Importance of cholesterol-rich membrane microdomains in the interaction of the S protein of SARS-coronavirus with the cellular receptor angiotensin-converting enzyme 2. Virology (2008) 381(2):215–21. doi: 10.1016/j.virol.2008.08.026 PMC7103374 18814896

[47] Nizet TA van den Elshout FJ Heijdra YF van de Ven MJ Mulder PG Folgering HT. Survival of chronic hypercapnic COPD patients is predicted by smoking habits, comorbidity, and hypoxemia. Chest (2005) 127(6):1904–10. doi: 10.1378/chest.127.6.1904 15947301

[48] Mahamat-Saleh Y Fiolet T Rebeaud ME Mulot M Guihur A El Fatouhi D. Diabetes, hypertension, body mass index, smoking and COVID-19-related mortality: a systematic review and meta-analysis of observational studies. BMJ Open (2021) 11(10):e052777. doi: 10.1136/bmjopen-2021-052777 PMC8557249 34697120

[49] Smith JC Sausville EL Girish V Yuan ML Vasudevan A John KM. Cigarette smoke exposure and inflammatory signaling increase the expression of the SARS-CoV-2 receptor ACE2 in the respiratory tract. Dev Cell (2020) 53(5):514–29 e3. doi: 10.1016/j.devcel.2020.05.012 32425701 PMC7229915

[50] Li L Wang X. Ferroptosis-associated cholesterol metabolism regulated by p85α in human bronchial epithelial cells with smoking. Clin Trans Discovery (2022) 2(1):e30. doi: 10.1002/ctd2.30

[51] Shang J Ye G Shi K Wan Y Luo C Aihara H. Structural basis of receptor recognition by SARS-CoV-2. Nature (2020) 581(7807):221–4. doi: 10.1038/s41586-020-2179-y PMC7328981 32225175

[52] Lan J Ge J Yu J Shan S Zhou H Fan S. Structure of the SARS-CoV-2 spike receptor-binding domain bound to the ACE2 receptor. Nature (2020) 581(7807):215–20. doi: 10.1038/s41586-020-2180-5 32225176

[53] Goel A Pinckney RG Littenberg B. APACHE II predicts long-term survival in COPD patients admitted to a general medical ward. J Gen Internal Med (2003) 18(10):824–30. doi: 10.1046/j.1525-1497.2003.20615.x PMC1494923 14521645

[54] Groenewegen KH Schols AM Wouters EF. Mortality and mortality-related factors after hospitalization for acute exacerbation of COPD. Chest (2003) 124(2):459–67. doi: 10.1378/chest.124.2.459 12907529

[55] Martin TR Lewis SW Albert RK. The prognosis of patients with chronic obstructive pulmonary disease after hospitalization for acute respiratory failure. Chest (1982) 82(3):310–4. doi: 10.1378/chest.82.3.310 7105857

[56] Laserna E Sibila O Aguilar PR Mortensen EM Anzueto A Blanquer JM. Hypocapnia and hypercapnia are predictors for ICU admission and mortality in hospitalized patients with community-acquired pneumonia. Chest (2012) 142(5):1193–9. doi: 10.1378/chest.12-0576 PMC3494472 22677348

[57] Vonderbank S Gibis N Schulz A Boyko M Erbuth A Gurleyen H. Hypercapnia at hospital admission as a predictor of mortality. Open Access Emerg Med (2020) 12:173–80. doi: 10.2147/OAEM.S242075 PMC7326210 32617025

[58] Zou X Chen K Zou J Han P Hao J Han Z. Single-cell RNA-seq data analysis on the receptor ACE2 expression reveals the potential risk of different human organs vulnerable to 2019-nCoV infection. Front Med (2020) 14(2):185–92. doi: 10.1007/s11684-020-0754-0 PMC7088738 32170560

[59] Nikiforuk AM Kuchinski KS Twa DDW Lukac CD Sbihi H Basham CA. The contrasting role of nasopharyngeal angiotensin converting enzyme 2 (ACE2) transcription in SARS-CoV-2 infection: A cross-sectional study of people tested for COVID-19 in British Columbia, Canada. EBioMedicine (2021) 66:103316. doi: 10.1016/j.ebiom.2021.103316 33819740 PMC8016616

[60] Casalino-Matsuda SM Nair A Beitel GJ Gates KL Sporn PH. Hypercapnia inhibits autophagy and bacterial killing in human macrophages by increasing expression of Bcl-2 and Bcl-xL. J Immunol (2015) 194(11):5388–96. doi: 10.4049/jimmunol.1500150 PMC4433787 25895534

[61] Jimenez JV Ackrivo J Hsu JY Wilson MW Labaki WW Hansen-Flaschen J. Lowering PCO2 with non-invasive ventilation is associated with improved survival in chronic hypercapnic respiratory failure. Respir Care (2023). doi: 10.4187/respcare.10813 PMC10676248 37137711

[62] Kohnlein T Windisch W Kohler D Drabik A Geiseler J Hartl S. Non-invasive positive pressure ventilation for the treatment of severe stable chronic obstructive pulmonary disease: a prospective, multicentre, randomised, controlled clinical trial. Lancet Respir Med (2014) 2(9):698–705. doi: 10.1016/S2213-2600(14)70153-5 25066329

[63] Murphy PB Rehal S Arbane G Bourke S Calverley PMA Crook AM. Effect of home noninvasive ventilation with oxygen therapy vs oxygen therapy alone on hospital readmission or death after an acute COPD exacerbation: A randomized clinical trial. JAMA (2017) 317(21):2177–86. doi: 10.1001/jama.2017.4451 PMC5710342 28528348

[64] Li GM Li YG Yamate M Li SM Ikuta K. Lipid rafts play an important role in the early stage of severe acute respiratory syndrome-coronavirus life cycle. Microbes Infect (2007) 9(1):96–102. doi: 10.1016/j.micinf.2006.10.015 17194611 PMC7110773

[65] Lu Y Liu DX Tam JP. Lipid rafts are involved in SARS-CoV entry into Vero E6 cells. Biochem Biophys Res Commun (2008) 369(2):344–9. doi: 10.1016/j.bbrc.2008.02.023 PMC7092920 18279660

[66] Nardacci R Colavita F Castilletti C Lapa D Matusali G Meschi S. Evidences for lipid involvement in SARS-CoV-2 cytopathogenesis. Cell Death Dis (2021) 12(3):263. doi: 10.1038/s41419-021-03527-9 33712574 PMC7952828

[67] Dias SSG Soares VC Ferreira AC Sacramento CQ Fintelman-Rodrigues N Temerozo JR. Lipid droplets fuel SARS-CoV-2 replication and production of inflammatory mediators. PloS Pathog (2020) 16(12):e1009127. doi: 10.1371/journal.ppat.1009127 33326472 PMC7773323

[68] Vadasz I Dada LA Briva A Trejo HE Welch LC Chen J. AMP-activated protein kinase regulates CO2-induced alveolar epithelial dysfunction in rats and human cells by promoting Na,K-ATPase endocytosis. J Clin Invest (2008) 118(2):752–62. doi: 10.1172/JCI29723 PMC2176184 18188452

[69] Vohwinkel CU Lecuona E Sun H Sommer N Vadasz I Chandel NS. Elevated CO(2) levels cause mitochondrial dysfunction and impair cell proliferation. J Biol Chem (2011) 286(43):37067–76. doi: 10.1074/jbc.M111.290056 PMC3199454 21903582

[70] Briva A Vadasz I Lecuona E Welch LC Chen J Dada LA. High CO2 levels impair alveolar epithelial function independently of pH. PloS One (2007) 2(11):e1238. doi: 10.1371/journal.pone.0001238 18043745 PMC2077933

[71] Amemiya-Kudo M Shimano H Hasty AH Yahagi N Yoshikawa T Matsuzaka T. Transcriptional activities of nuclear SREBP-1a, -1c, and -2 to different target promoters of lipogenic and cholesterogenic genes. J Lipid Res (2002) 43(8):1220–35. doi: 10.1194/jlr.M100417-JLR200 12177166

[72] Lee W Ahn JH Park HH Kim HN Kim H Yoo Y. COVID-19-activated SREBP2 disturbs cholesterol biosynthesis and leads to cytokine storm. Signal Transduct Target Ther (2020) 5(1):186. doi: 10.1038/s41392-020-00292-7 32883951 PMC7471497

[73] Hong EH Song JH Kang KB Sung SH Ko HJ Yang H. Anti-influenza activity of betulinic acid from zizyphus jujuba on influenza A/PR/8 virus. Biomol Ther (Seoul) (2015) 23(4):345–9. doi: 10.4062/biomolther.2015.019 PMC4489829 26157551

[74] Theo A Masebe T Suzuki Y Kikuchi H Wada S Obi CL. Peltophorum africanum, a traditional South African medicinal plant, contains an anti HIV-1 constituent, betulinic acid. Tohoku J Exp Med (2009) 217(2):93–9. doi: 10.1620/tjem.217.93 19212101

[75] Pavlova NI Savinova OV Nikolaeva SN Boreko EI Flekhter OB. Antiviral activity of betulin, betulinic and betulonic acids against some enveloped and non-enveloped viruses. Fitoterapia (2003) 74(5):489–92. doi: 10.1016/S0367-326X(03)00123-0 12837369

[76] Zapatero-Belinchon FJ Moeller R Lasswitz L van Ham M Becker M Brogden G. Fluvastatin mitigates SARS-CoV-2 infection in human lung cells. iScience (2021) 24(12):103469. doi: 10.1016/j.isci.2021.103469 34812415 PMC8599137

[77] Alqahtani JS Oyelade T Aldhahir AM Alghamdi SM Almehmadi M Alqahtani AS. Prevalence, Severity and Mortality associated with COPD and Smoking in patients with COVID-19: A Rapid Systematic Review and Meta-Analysis. PloS One (2020) 15(5):e0233147. doi: 10.1371/journal.pone.0233147 32392262 PMC7213702

[78] Chen Z Wen L Martin M Hsu CY Fang L Lin FM. Oxidative stress activates endothelial innate immunity via sterol regulatory element binding protein 2 (SREBP2) transactivation of microRNA-92a. Circulation (2015) 131(9):805–14. doi: 10.1161/CIRCULATIONAHA.114.013675 PMC4351177 25550450

[79] Sonett J Goldklang M Sklepkiewicz P Gerber A Trischler J Zelonina T. A critical role for ABC transporters in persistent lung inflammation in the development of emphysema after smoke exposure. FASEB J (2018) 32(12):fj201701381. doi: 10.1096/fj.201701381 29906247 PMC6219826

[80] Liu A Zhang X Li R Zheng M Yang S Dai L. Overexpression of the SARS-CoV-2 receptor ACE2 is induced by cigarette smoke in bronchial and alveolar epithelia. J Pathol (2021) 253(1):17–30. doi: 10.1002/path.5555 32991738 PMC7537258

[81] Kuo CW Su PL Huang TH Lin CC Chen CW Tsai JS. Cigarette smoke increases susceptibility of alveolar macrophages to SARS-CoV-2 infection through inducing reactive oxygen species-upregulated angiotensin-converting enzyme 2 expression. Sci Rep (2023) 13(1):7894. doi: 10.1038/s41598-023-34785-6 37193781 PMC10185955

[82] Russo P Bonassi S Giacconi R Malavolta M Tomino C Maggi F. COVID-19 and smoking: is nicotine the hidden link? Eur Respir J (2020) 55(6):2001116. doi: 10.1183/13993003.01116-2020 32341101 PMC7236819

[83] Jacobo-Albavera L Dominguez-Perez M Medina-Leyte DJ Gonzalez-Garrido A Villarreal-Molina T. The role of the ATP-binding cassette A1 (ABCA1) in human disease. Int J Mol Sci (2021) 22(4):1593. doi: 10.3390/ijms22041593 33562440 PMC7915494

