# Supplementary material for: Hypercapnia increases ACE2 expression and pseudo-SARS-CoV-2 entry in bronchial epithelial cells by augmenting cellular cholesterol
Source: Front Immunol. 2023 Oct 12;14:1251120. doi: 10.3389/fimmu.2023.1251120 (PMC10600497; doi:10.3389/fimmu.2023.1251120)
Supplement: Supplementary file 1 [file DataSheet_1.pdf]

Supplementary Material

Hypercapnia Increases ACE2 Protein Expression and Pseudo-SARS-CoV-2 Entry in Bronchial Epithelial Cells by Augmenting Cellular Cholesterol

Fei Chen, Aiko Matsuda, G. R. Scott Budinger, Peter H. S. Sporn, S. Marina Casalino-Matsuda*

*** Correspondence:** marinamatsuda@northwestern.edu

Supplementary Figures

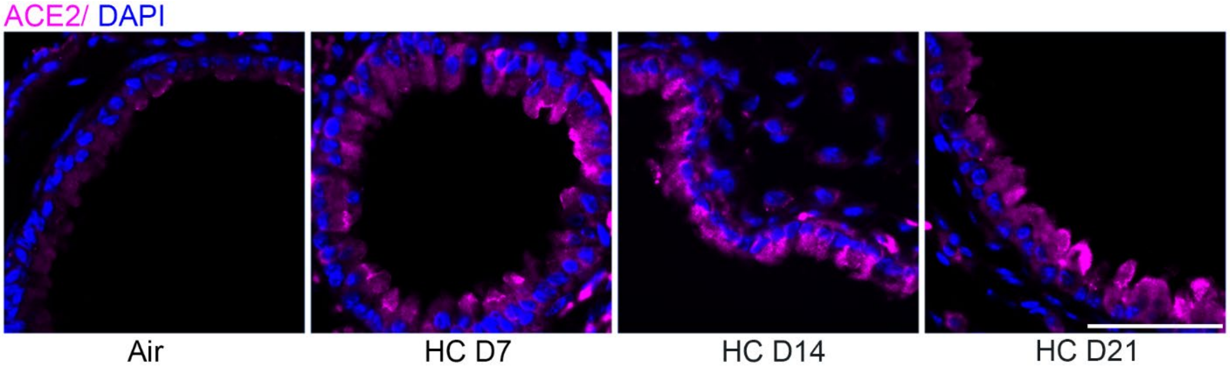

Supplementary Figure 1. Hypercapnia increases ACE2 protein expression in murine bronchial epithelium. ACE2 protein expression was assessed by IF in lungs of mice breathing ambient air or normoxic hypercapnia for 7 (HC D7), 14 (HC 14) and 21 (HC D21) days. Nuclei were stained with DAPI. Scale bar = 50 µM.

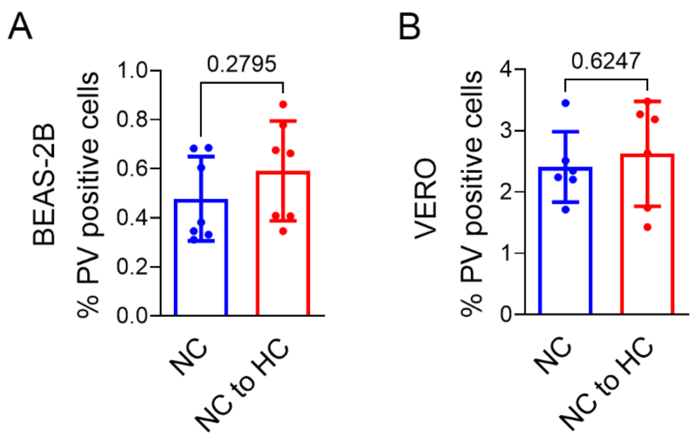

Supplementary Figure 2. Hypercapnia does not increase GFP fluorescence intensity in cells previously infected with Pseudo-SARS-CoV-2 in normocapnia. BEAS-2B (A) and VERO (B) cells were infected with pseudo-SARS-CoV-2 (PV) in normocapnia (NC), washed after 2 h to remove residual cell-free PV, cultured for an additional 18 h in NC or hypercapnia (HC), then fixed. Viral entry was assessed by IF and quantified as the percentage of PV-positive cells. Nuclei were stained with DAPI. All data are means ± SD. P values for comparisons between groups are shown.

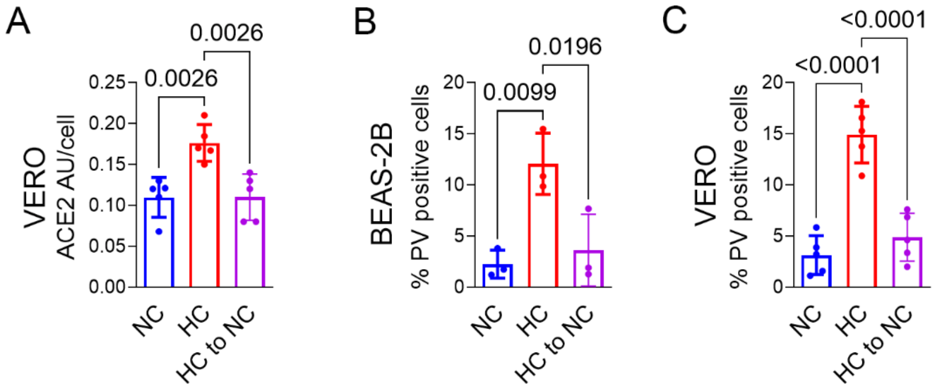

Supplementary Figure 3. Return to normocapnia reverses hypercapnia-induced increases in ACE2 expression and Pseudo-SARS-CoV-2 entry into epithelial cells. VERO (A, C) and BEAS-2B (B) cells were exposed to NC or HC for 2 days, pseudo-SARS-CoV-2 (PV) or vehicle were added, cells were cultured for an additional day in NC or HC, respectively, and then fixed. Alternatively, cells were pre-exposed to HC for 2 days, PV was added, then the cultures were switched to NC for 1 day (HC to NC) prior to fixation. ACE2 expression was assessed by IF and quantified as relative fluorescence intensity per cell, expressed in arbitrary units (AU) (A). Viral entry was assessed by IF and quantified as the percentage of PV-positive cells in BEAS-2B (B) and VERO (C) cultures. Nuclei were stained with DAPI. All data are means ± SD. P values for comparisons between groups are shown.

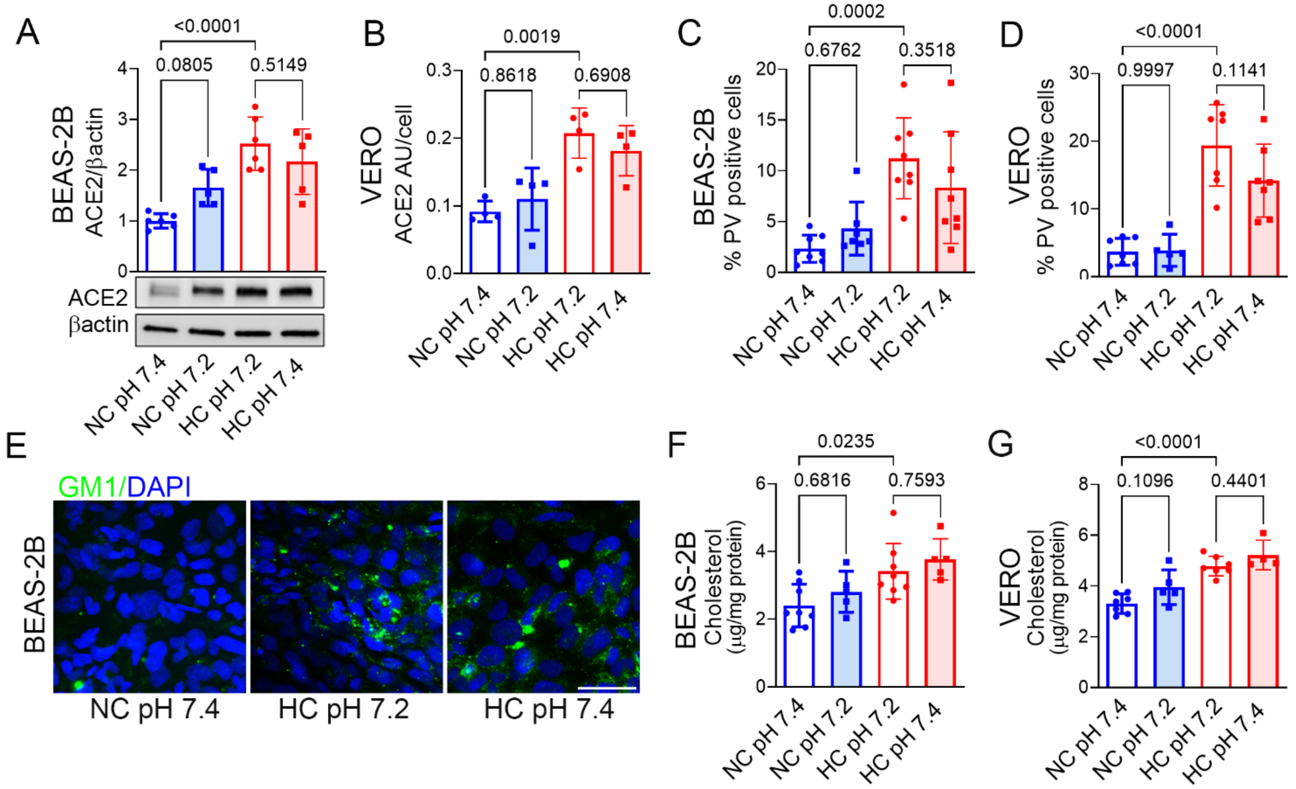

<u>Supplementary Figure 4</u>. Hypercapnia increases ACE2 protein expression, Pseudo-SARS-CoV-2 entry, and cholesterol accumulation in epithelial cells independently of extracellular acidosis. BEAS-2B and VERO cells were cultured in NC or HC for 2 days, pseudo-SARS-CoV-2 (PV) or vehicle were added, cells were cultured for an additional day in NC or HC, respectively, and then fixed. Tris base was added to media so that pH was maintained at 7.4 in HC (15% CO_2) or lowered to 7.2 in NC (5% CO_2). ACE2 was assessed by immunoblot in BEAS-2B cells (A) and quantified in VERO cells by IF as relative fluorescence intensity, expressed as arbitrary units (AU) (B). ACE2 was measured in in VERO (B). Viral entry was assessed by IF and quantified as the percentage of PV-positive cells in BEAS-2B (C) and VERO (D) cultures. Cholesterol accumulation was assessed in lipid rafts by GM1 staining in BEAS-2B cells (E) and by Amplex red assay in BEAS-2B (F) and VERO (G) cell lysates. β-actin was used as loading control in immunoblots. Nuclei were stained with DAPI. Scale bars = 50 µM. All data are means ± SD. P values for comparisons between groups are shown.

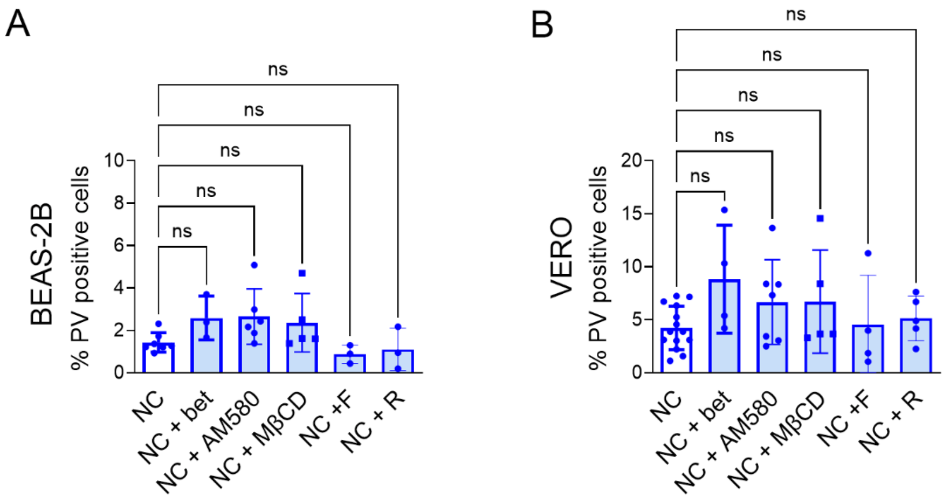

<u>Supplementary Figure 5</u>. **Inhibitors do not decrease Pseudo-SARS-CoV-2 entry into epithelial cells cultured in normocapnia.** BEAS-2B or VERO cells were treated with SREBP2 inhibitors betulin (bet, 7.5 µM) or AM580 (20 µM), the cholesterol depleting agent methyl-β-cyclodextrin (MβCD, 100 µM), or the statins fluvastatin (F, 50 nM) or rosuvastatin (R, 0.5 µM) for 3 days prior to addition of pseudo-SARS-CoV-2 virus (PV). Cells were cultured for an additional day in NC, then fixed. Viral entry was assessed by IF and quantified as the percentage of PV-positive cells in BEAS-2B (A) and VERO (B) cultures. All data are means ± SD. Differences between groups were not statistically significant (ns).

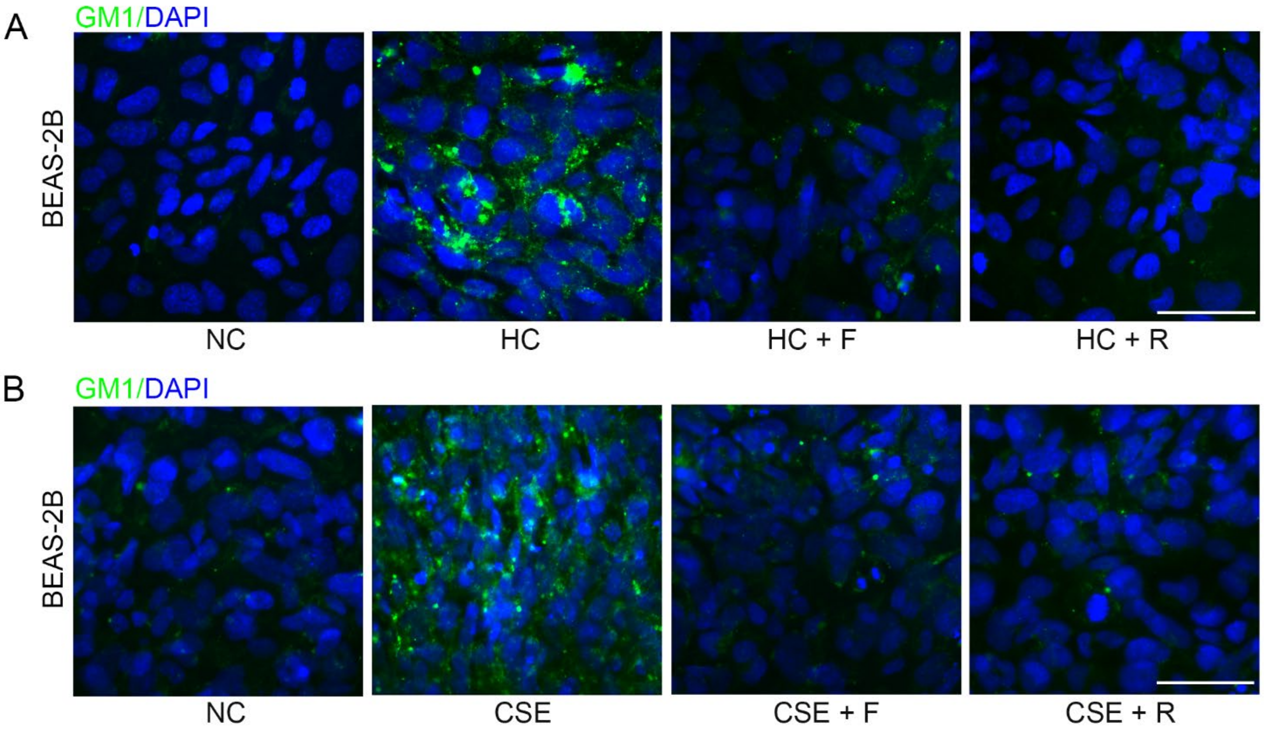

<u>Supplementary Figure 6</u>. **Statins block hypercapnia- and CSE-induced increases in lipid raft cholesterol in epithelial cells.** BEAS-2B cells were treated with fluvastatin (F, 50 nM) or rosuvastatin (R, 0.5 μM) for 1 day prior to and during exposure to CSE (1 μg/ml) for 2 days in NC or HC, pseudo-SARS-CoV-2 virus (PV) or vehicle were then added, cells were cultured for an additional day in NC or HC, respectively, then fixed and stained. Cholesterol accumulation in lipid rafts was assessed by labeling GM1 (A, B). Nuclei were labeled DAPI. Scale bars = 50 μM.

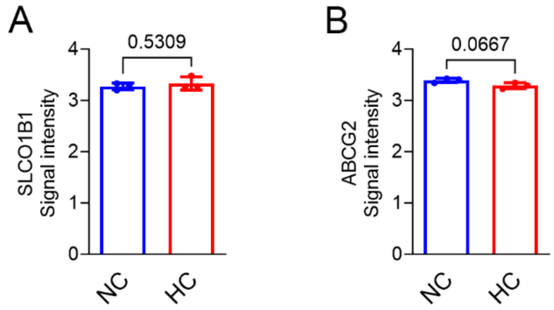

<u>Supplementary Figure 7</u>. **Hypercapnia does not change expression of statin transporters in epithelial cells.** HBE cells were exposed to NC or HC for 24 h, after which global gene expression was analyzed using Affymetrix GeneChip Hybridization. Gene expression for *SLCO1B1* (A) and *ABCG2* (B) was measured as signal intensity (n = 3). All data are means ± SD. P values for comparisons between groups are shown.
